# Genetic evaluation of cardiomyopathies in Qatar identifies enrichment of pathogenic sarcomere gene variants and possible founder disease mutations in the Arabs

**DOI:** 10.1002/mgg3.1709

**Published:** 2021-06-17

**Authors:** Kholoud N. Al‐Shafai, Mohammed Al‐Hashemi, Chidambaram Manickam, Rania Musa, Senthil Selvaraj, Najeeb Syed, Fazulur Vempalli, Muneera Ali, Magdi Yacoub, Xavier Estivill

**Affiliations:** ^1^ College of Health and Life Sciences Hamad Bin Khalifa University Doha Qatar; ^2^ Sidra Research Department Sidra Medicine Doha Qatar; ^3^ The Heart Hospital Hamad Medical Corporation Doha Qatar; ^4^ National Heart and Lung Institute Imperial College London London UK; ^5^ Quantitative Genomics Laboratories (qGenomics Barcelona Spain

**Keywords:** cardiomyopathy, targeted sequencing, genetic variants, Qatar

## Abstract

**Background:**

Hypertrophic cardiomyopathy (HCM) and dilated cardiomyopathy (DCM) are serious inherited heart diseases with various causative mutations identified. The full spectrum of causative mutations remains to be discovered, especially in understudied populations.

**Methods:**

Here, we established the DOHA Registry and Biobank for cardiomyopathies in Qatar, followed by sequencing of 174 genes on 51 HCM and 53 DCM patients, and 31 relatives.

**Results:**

In HCM, the analysis of 25 HCM‐associated genes showed that 20% of HCM cases had putative pathogenic variants for cardiomyopathy, mainly in sarcomere genes. Additional 49% of HCM cases had variants of uncertain significance, while 31% of HCM cases had likely benign variant(s) or had no variants identified within the analyzed HCM genes. In DCM, 56 putative DCM genes were analyzed. Eight percent of DCM cases had putative pathogenic variants for DCM, in the *TTN* gene while 70% of cases had variants of uncertain significance, in the analyzed DCM genes, that will need further pathogenicity assessment. Moreover, 22% of DCM cases remain unexplained, by having likely benign variant(s) or having no variants detected in any of the analyzed DCM genes.

**Conclusion:**

We identified or replicated at least four recurrent variants among cardiomyopathy patients, which could be founder disease mutations in the Arabic population, including a frameshift variant (c.1371_1381dupTATCCAGTTAT) of unknown significance in the *FKTN* gene which seems to cause DCM in homozygosity, and HCM in heterozygosity. *In vivo* and/or *in vitro* functional validation need to be pursued in order to assess the pathogenicity of the identified variants.

## Graphical abstract

We established the DOHA Registry and Biobank for cardiomyopathies in Qatar, followed by sequencing of 174 genes on 51 HCM and 53 DCM patients, and 31 relatives. Twenty percent of HCM cases and eight% of DCM cases had pathogenic variants for cardiomyopathy, while 31% of HCM cases and 22% of DCM cases remain unexplained by having likely benign variant(s) or having no variants detected in any of the analysed genes.We replicated at least four recurrent variants among cardiomyopathy patients, which could be founder disease mutations in the Arabic population, including a frameshift variant (c.1371_1381dupTATCCAGTTAT) in the *FKTN* gene which seems to cause DCM in homozygosity and HCM, in heterozygosity.

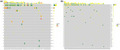

## 1 INTRODUCTION

Cardiovascular disorders (CVD) encompass a wide range of heart diseases extending from a myocardial infraction to congenital heart diseases, most of which are heritable. These conditions include cardiomyopathies (CM), which are a monogenic set of CVDs characterized by structural and functional alterations of the myocardium in the absence of disorders, such as coronary heart disease, hypertension, and abnormalities, such as valve or congenital heart defects (Yacoub, 2014). CM is a major cause for sudden cardiac death and heart failure. It has been estimated that nearly 50% of patients who die suddenly during childhood or adolescence or that undergo heart transplantation have CM (McKenna et al., 2017). Primary inherited cardiomyopathy types include hypertrophic cardiomyopathy (HCM), dilated cardiomyopathy (DCM), arrhythmogenic right ventricular cardiomyopathy (ARVC), and restrictive cardiomyopathy (RCM). Even with the current advances in surgical treatment and disease management, CM are considered severe life‐threatening conditions that compromise seriously the life of people suffering from these disorders.

In terms of etiology, CM cluster in families and are genetically heterogeneous. Fifty percent of HCM cases (Marian, 2010) and between 30–50% of DCM cases are familial (Yacoub, 2014). So far, at least 100 genes are known to be implicated in the pathogenesis of CM (McNally et al., 2015). This includes genes encoding for the sarcomere, desmosome, cytoskeleton, and ion‐homeostasis related proteins (McNally et al., 2015; Tobita et al., 2018). Most of the identified CM genetic variants are inherited in an autosomal dominant manner (McKenna et al., 2017), with some ethnic‐specific founder mutations (Adalsteinsdottir et al., 2014; Vattulainen et al., 2015) though with variable expressivity and penetrance (Keeling et al., 1995). Identifying the full spectrum of the heritable components of CM is one of the major challenges of cardiovascular genetic research, especially in understudied populations like the citizens of Qatar.

In this study, we have explored the genetic basis of CM in the population of Qatar through the establishment of The Dilated cardiomyopathy, Obstructive‐Hypertrophic cardiomyopathy and Arrhythmogenic right ventricular cardiomyopathy Registry (The DOHA‐Registry) and Biobank at the Heart Hospital in Qatar, followed by genetic testing for the registry participants. The study took into account unique features of the population of Qatar, including the high level of consanguinity estimated at 54% (Bener & Alali, 2006) which increases the burden of recessively inherited diseases including heart diseases (Aburawi et al., 2015). In addition, the endogamous nature of the populations living in Qatar, including Qataris, other Arab populations, and Southeast Asians, can lead to autozygosity of founder mutations, and thus helps revealing novel disease mutations and genes that are not detected in outbred populations (Maddirevula et al., 2019).

## 2 MATERIALS AND METHODS

### 2.1 Ethical compliance

This study was approved by the institutional review board (IRB) of the participating institutions and was conducted according to the guidelines of the Declaration of Helsinki. Informed consent/assent was obtained from all the study subjects.

### 2.2 Study subjects

Fifty‐one unrelated HCM index cases and 53 unrelated non‐ischemic DCM index cases were enrolled to The DOHA‐Registry and Biobank, at the Heart Hospital in Qatar, in addition to 31 of their relatives (4 affected, 12 unaffected, and 15 asymptomatic) (Pedigrees of informative HCM and DCM families are provided in Figure S1). Disease diagnosis was defined according to the commonly used WHO/International Society of Federation of Cardiology Task Force clinical ("Anon.," 1980). Patients were considered to have familial cardiomyopathy if the proband had at least one additional affected family member with any type of cardiomyopathy or if the proband had a family history of sudden cardiac death.

For each enrolled subject, basic demographic and family history data were collected along with the clinical data of the performed clinical examinations, including electrocardiogram (ECG), echocardiogram (Echo), and magnetic resonance imaging (MRI). For genetic investigation, 10–20 ml (depending on the age of the participant) of whole blood in EDTA tubes were obtained from each study subject.

### 2.3 Generation of targeted sequencing data

For all study subjects, genomic DNA was extracted from whole blood (Qiagen, Netherlands) and validated in terms of quality and quantity using standard techniques. The TruSight Cardio sequencing kit from illumina (illumina Inc) was used to prepare libraries that are enriched for the coding and flanking intronic boundaries of 174 genes known to be involved in inherited cardiac conditions (ICCs) (Pua et al., 2016) (Table S1). DNA sequencing was performed on illumina Miseq instrument (illumina Inc) using either v2 or v3 chemistry kit to produce 300‐bp paired‐end sequencing reads.

### 2.4 Sequence alignment and variant calling

After demultiplexing the raw sequencing files (.bcl files) into separate FastQ files, the quality of sequencing reads was assessed using FASTQC (Andrews, 2010). Adaptor sequences were trimmed using trimadap (https://github.com/lh3/trimadap) and high‐quality reads were mapped to the NCBI human reference genome CRGh37/hg19 using Burrows‐Wheeler Aligner (BWA) v0.7.8 particularly BWA‐MEM algorithm (arXiv:1303.3997[q‐bio‐GN]). Duplicate reads after alignment were marked by SAMBLASTER (Faust & Hall, 2014), and BAM files were sorted using SAMtools (Li et al., 2009). These steps were accomplished simultaneously using BWA‐kit package (Li, 2013). After the alignment, reads were recalibrated and variants were called using the Genome Analysis Toolkit (GATK) v4.1 (McKenna et al., 2010). Joint calling was performed on all samples to generate a single VCF for all samples. Variant Effect predictor (VEP) (Ruffier et al., 2017) was used to annotate the VCF file and Loss‐Of‐Function Transcript Effect Estimator (LOFTEE) (https://github.com/konradjk/loftee) plugin was used to annotate Loss of Function Variants (LoF). Vcfanno (Pedersen et al., 2016) was used to annotate VCF file with extensive available data resources including GNOMAD (Karczewski et al., 2019), EXAC (Lek et al., 2016), TOPMED (Taliun et al., 2019), and 1K genome (The Genomes Project et al., 2015), in addition to CADD (Rentzsch et al., 2019) and GERP (Cooper et al., 2005) scores. We have compared the allele frequency of our variants with other region‐specific datasets from the Greater Middle East (Scott et al., 2016) and Qatar (Fakhro et al., 2016). Custom Python scripts were written and used to filter data and generate results in a specific user‐friendly format.

Any variant with a detected coverage of less than 20‐fold was excluded. Intronic variants and synonymous variants were excluded, along with variants with low or modifier impact, based on SnpEff (Cingolani et al., 2012). Variants of minor allele frequency greater than 0.01 in any of the freely available control databases were filtered out, along with variants of CADD prediction score (Rentzsch et al., 2019) of less than 13. Moreover, we further focused our variants analysis by including variants observed in HCM and DCM genes only. This included 25 genes that are associated with the HCM phenotype (Ingles et al., 2019) and 56 putative DCM genes of the TruSight Cardio sequencing panel (Mazzarotto et al., 2020) (Table S1).

**FIGURE 1:**
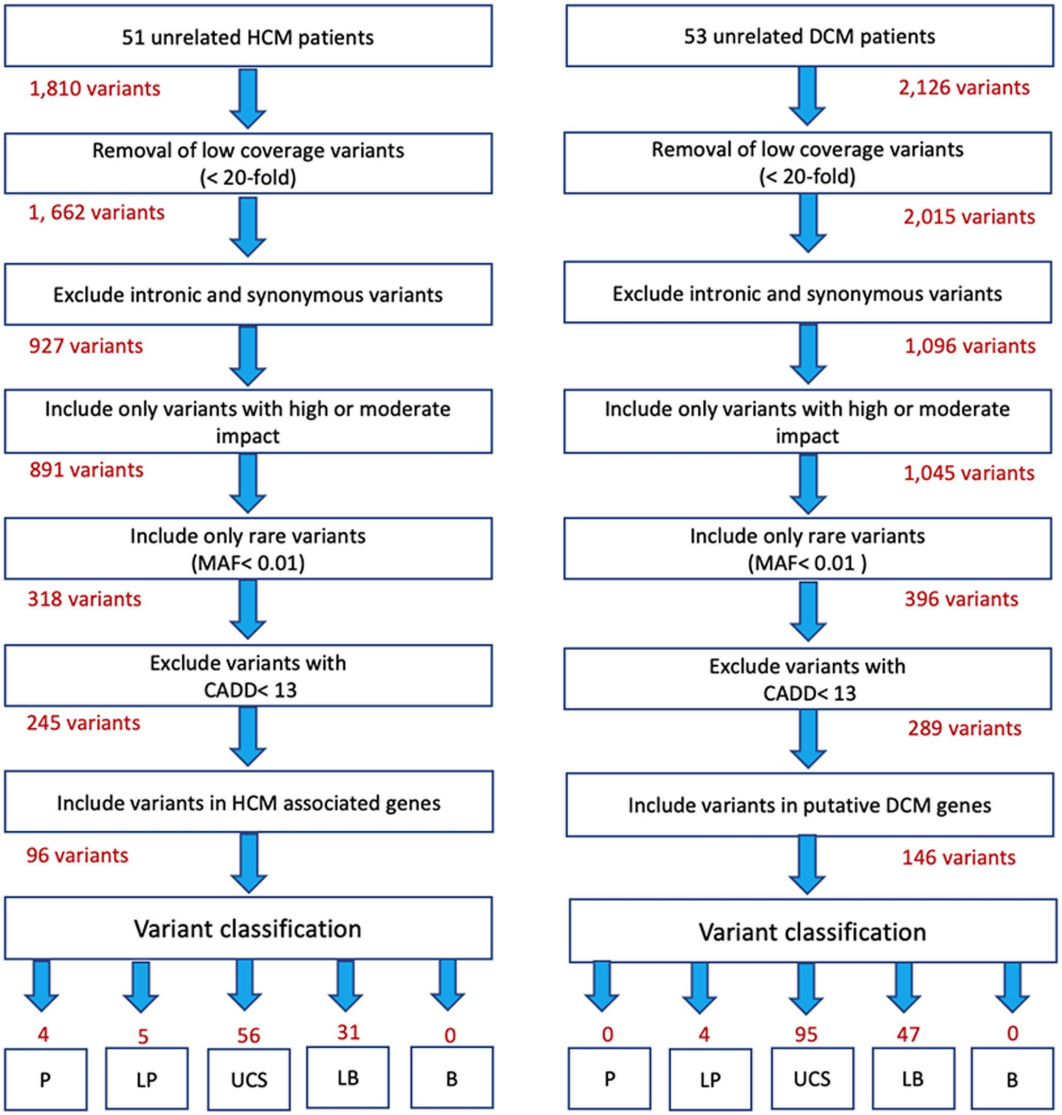
Summary of variant filtration and classification. The number of variants is given for each step. B, benign; CADD, combined annotation‐dependent depletion; DCM, dilated cardiomyopathy; HCM, hypertrophic cardiomyopathy; LB, likely benign; LP, likely pathogenic; MAF, minor allele frequency; P, pathogenic; UCS, uncertain significance

The variants in these targeted genes were classified according to The American College of Medical Genetics and Genomics (ACMG) and the Association for Molecular Pathology (AMP) guidelines (Richards et al., 2015) into five classifications which are “pathogenic,” “likely pathogenic,” “uncertain significance,” “likely benign,” and “benign” (Figure 1). Variants were classified using the available online variant interpretation tools including CardioVAI (Nicora et al., 2018) or CardioClassifier (Whiffin et al., 2018), or manually after assessing the available evidence in each criteria of the ACMG‐AMP guidelines.

## 3 RESULTS

### 3.1 The Doha Registry characteristics

From the Doha Registry, we analyzed 51 unrelated HCM patients and 53 unrelated non‐ischemic DCM patients. The basic and clinical features of the study subjects are summarized in Table 1. For HCM cases, the mean age was 48 ± 14 years, 46 patients (90%) were males and 26 (51%) were Arabs. A family history of heart disease or sudden cardiac death was present in 19 patients, and 9 (18%) had atrial fibrillation. For DCM cases (n = 53), the mean age was 52 ± 13 years, 43 patients (81%) were males and 20 (38%) were Arabs. A family history of heart diseases or sudden cardiac death was present in 8 patients, and 5 (9%) had atrial fibrillation.

**TABLE 1:** Basic and clinical features of the unrelated HCM (n=51) and DCM (n=53) index cases

	HCM index cases (n = 51)	DCM index cases (n = 53)
Age (years)	48 ± 14	52 ± 13
Gender		
Male (n)	46 (90%)	43 (81%)
Female (n)	5 (10%)	10 (19%)
Ethnicity		
Arabs	26 (51%)	20 (38%)
South Asians	21 (41%)	21 (40%)
Others (Iranians, Philipinos, Ghanaians, Naijirians Athiopians and Turkish)	4 (8%)	12 (22%)
Familial	19/40 (47.5%)	8/38 (21%)
Sporadic	21/40 (52.5%)	30/38 (79%)
Family history of heart disease and/or sudden death (n)	19/40 (47.5%)	8/38 (21%)
NYHA functional class of 1	37/47(79%)	32 (60%)
NYHA functional class ≥2	10/47 (21%)	21 (40%)
B‐type natriuretic peptide (pg/ml)	1723 ± 2270	8720 ± 50349
Atrial fibrillation	9/51 (18%)	5 (9%)
Non‐sustained VT	12/49 (24%)	10/48 (21%)
Echocardiography		
Interventricular septum (mm)	18.4 ± 5	0.9 ± 0.2
Mitral regurgitation ≥moderate	6/47 (13%)	16/53 (30%)
E/e′	13 ± 5	12 ± 5
Cardiopulmonary excersis testing (n = 13 HCM, 4 DCM)		
Rest exercise heart rate (beats/min)	68.8 ± 12.5	76 ± 26
Peak exercise heart rate (beats/min)	137 ± 17	145 ± 12
Rest exercise systolic blood pressure (mmHg)	127.2 ± 18	145 ± 21
Peak exercise systolic blood pressure (mmHg)	164 ± 36	201 ± 52
Treatment (n)		
Amiodarone	5 (10%)	6 (11%)
ICD implantation	8 (16%)	8 (15%)
Myectomy	5 (10%)	0 (0%)
Heart transplantation	1 (2%)	0 (0%)
Mortality	3/48 (6%)	3/48 (6%)

Values are n (%), the mean ± SD.

Abbreviations: DCM, dilated cardiomyopathy; HCM, hypertrophic cardiomyopathy; ICD, implantable cardioverter defibrillator; NYHA, New York Heart Association; VT, ventricular tachycardia.

### 3.2 Quality of the sequencing data

The DNA of the study subjects (n = 135) was sequenced for 174 cardiology genes. In the targeted regions, the median read depth was 86 and 80%–90% of the targeted regions had a read depth of over 20.

### 3.3 Genetic profile of HCM

For the 51 unrelated HCM cases, a total of 1,810 variants were identified including 1,757 single‐nucleotide variants (SNVs) and 53 insertion/deletion (indels) variants. The exclusion of low coverage variants (<20‐fold), intronic and synonymous variants along with variants of MAF >0.01% gave a total of 317 variants, 245 of them having a CADD score of >13.

Of the 245 variants, 96 variants were observed in 16 out of 25 HCM‐associated genes, in 43 HCM cases (Table S2a). Of these 96 variants, nine variants were putative pathogenic variants (classified as “pathogenic” or “likely pathogenic”) and were observed in ten HCM cases (20%) in sarcomere or sarcomere‐associated genes. Seven of the nine variants were in genes that have a definitive association with HCM including *MYBPC3*, *MYH7*, *TNNT2*, and *TNNI3*, while the remaining two variants were in *TTN* and *TRIM63* genes, which have limited association to HCM (Ingles et al., 2019).

Two of the nine putative pathogenic variants were novel variants (c.3009_3010delTC and c.51dupG) in *MYBPC3* (IGV screenshots are given in Figure S2). The frameshift variant (c.51dupG) in *MYBPC3* was seen in two unrelated Sri Lankan HCM patients and based on LOFTEE, is an LoF variant with a high confidence.

In addition to cases with putative pathogenic variants, 25 additional HCM cases (49%) had variant(s) of uncertain significance that need to be further evaluated. This include a novel variant (p.Ser57del) in *TTN* which was observed in a heterozygous state in a Qatari HCM patient (HCM3 family of Figure S1) (IGV screenshots are given in Figure S2).

In terms of variant zygosity, the variant p.Arg145Gln in *TNNI3* which was classified as a likely pathogenic variant and the variant p.Ala57Asp in *MYL3* which was classified as a variant of uncertain significance, were detected in a homozygous state, in a Jordanian (HCM4 family of Figure S2) and a Qatari HCM index patients, respectively.

**FIGURE 2:**
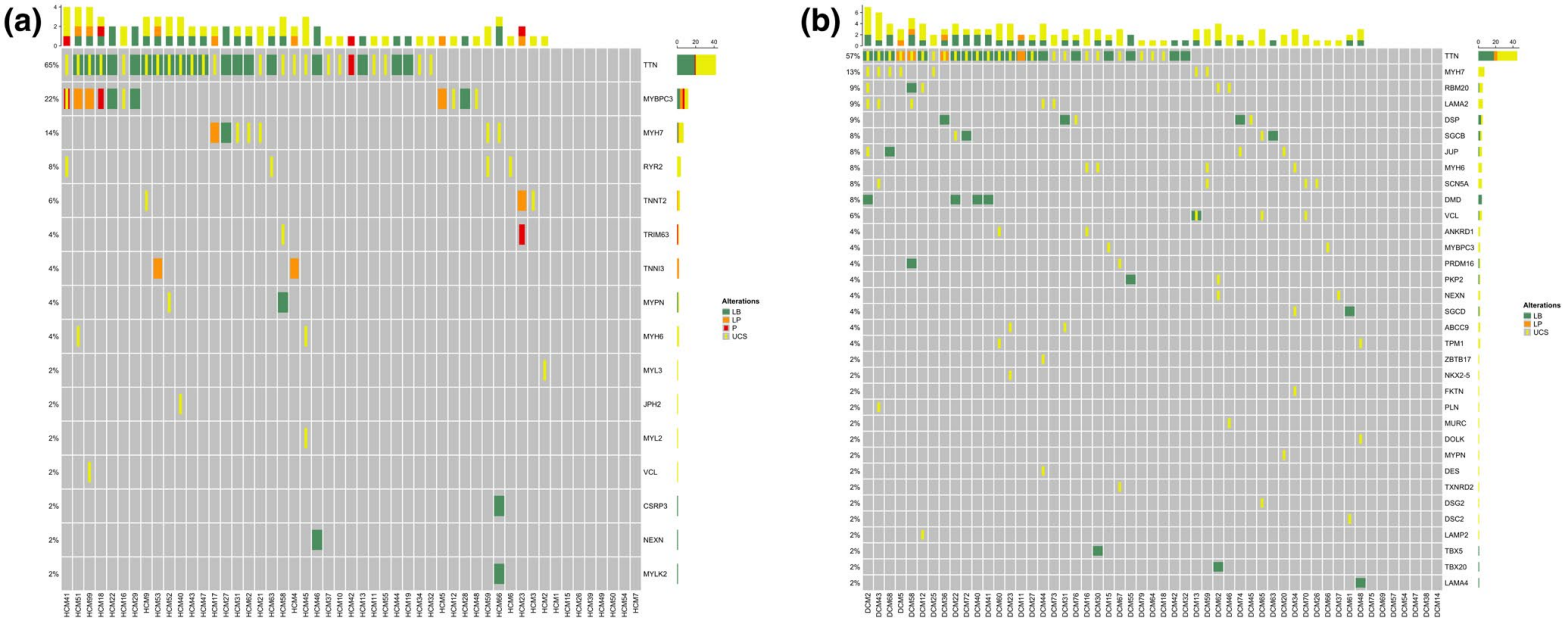
The genetic distribution of variants in HCM cases (a) and DCM cases (b), in genes linked to HCM and DCM, respectively, with respect to variant classification. Each column represents one patient and each row represents one gene. The higher bar at the top summarizes the variants seen in each of the columns

Though eight HCM cases (15%) had likely benign variants and additional eight HCM cases (15%) did not have any variants within the analyzed HCM genes. Figure 2a shows the genetic distribution of the detected variants in HCM cases in genes closely linked to HCM.

### 3.4 Genetic profile of DCM

For the 53 unrelated DCM cases, a total of 2,126 variants were identified, including 2060 single‐nucleotide variants (SNVs) and 66 insertion/deletion (indels) variants. The exclusion of low coverage variants, intronic and synonymous variants along with variants of MAF >0.01%, gave a total of 396 variants, 289 of them with a CADD score above 13 (Figure 1).

Out of the 289 variants, 146 variants were observed in 33 putative DCM genes in 45 DCM cases (Table S2b). Of the 146 variants, only four variants were putative pathogenic variants in *TTN*; a definitive causative gene for DCM, and were seen privately in four DCM cases (8%). Two of these four *TTN* variants were novel (not reported in any population datasets) (IGV screenshots are shown in Figure S2).

Moreover, additional 37 DCM cases (70%) had variants of uncertain significance including 50 variants observed in definitive genes for DCM (Mazzarotto et al., 2020). Of those 50 variants, 35 variants were in *TTN* gene (31 of them were missense variants). Moreover, two of the variants of uncertain significance were seen in a homozygous state. This include a variant (c.5769delG) in *MYH7* which was detected in a Sudanese DCM patient, and a variant (c.1371_1381dupTATCCAGTTAT) in *FKTN* which was seen in a Qatari DCM patient. Such variants would require further investigation in order to assess their role in disease phenotype.

Other 4 DCM cases (8%) had likely benign variants while the remaining eight DCM cases (15%) had no variants detected in any of the analyzed DCM genes. Figure 2b shows the genetic distribution of the detected variants in DCM cases in genes linked to DCM. The identified variants were submitted to the publicly available ClinVar Database and the accession numbers are provided in Table S3.

### 3.5 Overlap between HCM and DCM

Since there were 16 genes overlapping between the analyzed HCM and DCM genes (as given in Table S1), we checked for the overlapped variants in these genes between HCM and DCM cases. We observed four shared variants between HCM and DCM (shown in bold in Tables S2a and S2b). Three of the four variants were in *TTN* (two were likely benign variants and one was variant of unknown significance) while the fourth variant was of unknown significance in *MYH6*.

## 4 DISCUSSION

We present here the genetic basis of dilated and hypertrophic cardiomyopathy in Qatar through the establishment of the Doha Registry and Biobank at the Heart Hospital, and the sequencing of 174 genes known to be involved in inherited cardiac disorders followed by variant analysis restricted to HCM and DCM genes. Yet, the panel used in this study does not include *OBSCN and MYOM* genes, that are associated with HCM (Ingles et al., 2019).

We identified a total of 13 putative pathogenic variants in 10 HCM and four DCM cases, thus explaining the disease in 20% of HCM cases and eight percent of DCM cases. For HCM, all of the nine detected putative pathogenic variants were in sarcomere or sarcomere‐associated gene, with *MYBPC3* and *MYH7* variants overshadowed, consistent with previous reports (Biagini et al., 2014). In DCM, the four putative pathogenic variants were in *TTN*; a definitive causative gene for DCM. However, 16 HCM (33%) and 12 DCM cases (22%) had being or likely benign variant(s) or did not have any variants in any of the analyzed HCM or DCM genes. Hence, whole genome sequencing (WGS) should eventually be performed for those patients, in addition to their relatives, in order to reduce the major accompanied difficulties of determining the pathogenicity of the variants detected from WGS.

Moreover, we detected accumulation of variants in our patients. For example, HCM45, which has obstructive HCM and E/e′ value of 25, the highest value within our patients, had three variants of unknown significance in *MYL2*, *MYH6*, and *TTN* genes. The role of such variants requires further investigations since as reported previously, accumulation of disease mutations could contribute to the severity of the disease phenotypes (Tobita et al., 2018) and could shape the clinical heterogeneity seen in our cardiomyopathy cohort.

Segregation analysis of affected and unaffected relatives of each proband could help toward weighting the contribution of each variant. For example, the pathogenic variant (c.2905C>T, p.Gln969*) in *MYPBC3*, in HCM18 and the likely pathogenic variant (p.Arg295Cys) in *TNNT2*, in HCM23 were detected in unaffected family members of the patients carrying the variant(s), which could indicate incomplete penetrance of the variants and may reduce the possibility for those variants to be exclusively underlying disease pathogenesis (Figure S1). In the future, we plan to overcome the limitation of our study in terms of the small number of relatives and the limited informativity of our recruited families, by recruiting additional family members of our index cases. Also, it essential that those relatives undergo the required clinical examinations in order to confirm their disease state.

Moreover, the fact that the Arab populations are understudied, together with the endogamous nature of the Qatari population and the Arab and Southeast Asian communities living in Qatar, allowed us to identify novel variants, that were not detected in any outbred population and therefore are not present in other clinical databases.

In HCM, 19 novel variants were detected in *MYBPC3*, *NEXN*, *RYR2*, *TNNT2*, and *TTN* genes in 17 HCM cases. This includes two putative pathogenic variants (in *MYBPC3)*, 12 variants of unknown significance, in addition to four likely benign variants.

In addition, 25 novel variants were identified in 24 DCM cases, 12 of the variants were in the *TTN* gene, 2 of the 12 variants were likely pathogenic, three were likely benign and seven were variants of uncertain significance. Functional evaluation and the genetic data of a larger dataset, such as that of Qatar Genome Programme (QGP) (qatargenom.org.qa) could facilitate the further assessment of the role of these novel variants.

The high level of consanguinity in the studied populations led to the identification of rare variants in a homozygous status. For example, an 11 base‐pair insertion variant in *FKTN* (c.1371_1381dupTATCCAGTTAT), which was classified as variant of uncertain significance, was detected in homozygosity in a Qatari DCM patient. The same variant was seen, also in a homozygous status, in another Qatari DCM patient who died suddenly at age of 38 years old (from unpublished records of Hamad Medical Corporation, Qatar). The parents of the later DCM patient share the same surname, indicating consanguinity. The same variant was detected in one of our Qatari HCM patient, but in the heterozygous state. The same variant was reported in 2016 in relation to abnormalities in the cardiovascular system (Retterer et al., 2016). This variant in *FKTN*, associated with both HCM and DCM, is likely a Qatari/Arabic founder mutation leading to cardiomyopathy in both the heterozygous and homozygous state.

Homozygous putative pathogenic variants seem to be associated with severe disease phenotype. In HCM, for example, the likely pathogenic variant p.Arg145Gln in *TNNI3* is seen in a homozygous state in a Jordanian patient with apical hypertrophy, palpitations due to non‐sustained ventricular tachycardia, and an age of onset of 31 years old. The sister of this patient is also homozygous for the same variant and has apical hypertrophy with occasional shortness of breath on exertion with grade diastolic dysfunction and lower limb edema, and an age of onset of 34 years old.

Moreover, our study was able to replicate variants seen in other Arabic cardiomyopathy patients. For example, the likely pathogenic variant (p.Ala57Asp) in *MYL3* gene variant is seen in a homozygous state in a 44 years old Qatari HCM patient with obstructive hypertrophy, was reported earlier in a 49 years old Tunisian HCM patient (Jaafar et al., 2015) and interestingly both patients had atrial fibrillation along with the hypertrophy. Also, we found the variant (p.Glu619Lys) in *MYBPC3* in an Egyptian HCM patient and was reported in two other unrelated HCM patients from Egypt (Kassem et al., 2013). Also, in a Saudi HCM patient, we detected a *MYH7* gene variant (p.Arg1662His) which was reported earlier in an Egyptian HCM patient (Kassem et al., 2013).

In conclusion, we claim the need for systematic evaluation of the pathogenicity of the detected variants, including the assessment of possible compensatory effect of variants, in relation to the disease phenotype, especially for monogenic and life‐threatening diseases like cardiomyopathies.

## CONFLICT OF INTEREST

The authors declare that there is no conflict of interest related to this research.

## Supporting information

Table S1‐Fig S1‐S2

Table S2

Table S3

## Data Availability

The generated data from this study are available from the corresponding authors upon a justifiable request.

## References

[mgg31709-bib-0001] Aburawi, E. H., Aburawi, H. E., Bagnall, K. M., & Bhuiyan, Z. A. (2015). Molecular insight into heart development and congenital heart disease: An update review from the Arab countries. Trends in Cardiovascular Medicine, 25(4), 291–301. 10.1016/j.tcm.2014.11.007 25541328

[mgg31709-bib-0002] Adalsteinsdottir, B., Teekakirikul, P., Maron, B. J., Burke, M. A., Gudbjartsson, D. F., Holm, H., Stefansson, K., DePalma, S. R., Mazaika, E., McDonough, B., Danielsen, R., Seidman, J. G., Seidman, C. E., & Gunnarsson, G. T. (2014). Nationwide study on hypertrophic cardiomyopathy in Iceland: evidence of a MYBPC3 founder mutation. Circulation, 130(14), 1158–1167. 10.1161/circulationaha.114.011207 25078086

[mgg31709-bib-0003] Akinrinade, O., Ollila, L., Vattulainen, S., Tallila, J., Gentile, M., Salmenperä, P., Koillinen, H., Kaartinen, M., Nieminen, M. S., Myllykangas, S., Alastalo, T.‐P., Koskenvuo, J. W., & Heliö, T. (2015). Genetics and genotype–phenotype correlations in Finnish patients with dilated cardiomyopathy. European Heart Journal, 36(34), 2327–2337. 10.1093/eurheartj/ehv253 26084686 PMC4561350

[mgg31709-bib-0040] Anon. (1980). Report of the WHO/ISFC task force on the definition and classification of cardiomyopathies. British Heart Journal, 44(6), 672–673. 10.1136/hrt.44.6.672 7459150 PMC482464

[mgg31709-bib-0005] Bener, A., & Alali, K. A. (2006). Consanguineous marriage in a newly developed country: the Qatari population. Journal of Biosocial Science, 38(2), 239–246. 10.1017/S0021932004007060 16490156

[mgg31709-bib-0006] Biagini, E., Olivotto, I., Iascone, M., Parodi, M. I., Girolami, F., Frisso, G., Autore, C., Limongelli, G., Cecconi, M., Maron, B. J., Maron, M. S., Rosmini, S., Formisano, F., Musumeci, B., Cecchi, F., Iacovoni, A., Haas, T. S., Bacchi Reggiani, M. L., Ferrazzi, P., … Rapezzi, C. (2014). Significance of sarcomere gene mutations analysis in the end‐stage phase of hypertrophic cardiomyopathy. American Journal of Cardiology, 114(5), 769–776. 10.1016/j.amjcard.2014.05.065 25037680

[mgg31709-bib-0007] Cingolani, P., Platts, A., Wang, L. L., Coon, M., Nguyen, T., Wang, L., Land, S. J., Lu, X., & Ruden, D. M. (2012). A program for annotating and predicting the effects of single nucleotide polymorphisms, SnpEff: SNPs in the genome of Drosophila melanogaster strain w1118; iso‐2; iso‐3. Fly (Austin), 6(2), 80–92. 10.4161/fly.19695 22728672 PMC3679285

[mgg31709-bib-0008] Cooper, G. M., Stone, E. A., Asimenos, G., Green, E. D., Batzoglou, S., & Sidow, A. (2005). Distribution and intensity of constraint in mammalian genomic sequence. Genome Research, 15(7), 901–913. 10.1101/gr.3577405 15965027 PMC1172034

[mgg31709-bib-0009] Fakhro, K. A., Staudt, M. R., Ramstetter, M. D., Robay, A., Malek, J. A., Badii, R., Al‐Marri, A.‐N., Khalil, C. A., Al‐Shakaki, A., Chidiac, O., Stadler, D., Zirie, M., Jayyousi, A., Salit, J., Mezey, J. G., Crystal, R. G., & Rodriguez‐Flores, J. L. (2016). The Qatar genome: a population‐specific tool for precision medicine in the Middle East. Human Genome Variation, 3, 16016. 10.1038/hgv.2016.16. https://www.nature.com/articles/hgv201616#supplementary‐information 27408750 PMC4927697

[mgg31709-bib-0010] Faust, G. G., & Hall, I. M. (2014). SAMBLASTER: fast duplicate marking and structural variant read extraction. Bioinformatics (Oxford, England), 30(17), 2503–2505. 10.1093/bioinformatics/btu314 PMC4147885 24812344

[mgg31709-bib-0012] Ingles, J., Goldstein, J., Thaxton, C., Caleshu, C., Corty, E. W., Crowley, S. B., Dougherty, K., Harrison, S. M., McGlaughon, J., Milko, L. V., Morales, A., Seifert, B. A., Strande, N., Thomson, K., Peter van Tintelen, J., Wallace, K., Walsh, R., Wells, Q., Whiffin, N., … Funke, B. (2019). Evaluating the clinical validity of hypertrophic cardiomyopathy genes. Circulation: Genomic and Precision Medicine, 12(2), e002460. 10.1161/circgen.119.002460 30681346 PMC6410971

[mgg31709-bib-0013] Jaafar, N., Girolami, F., Zairi, I., Kraiem, S., Hammami, M., & Olivotto, I. (2015). Genetic profile of hypertrophic cardiomyopathy in Tunisia: Is it different? Global Cardiology Science and Practice, 2015(1), 16. 10.5339/gcsp.2015.16 26779504 PMC4448072

[mgg31709-bib-0014] Karczewski, K. J., Francioli, L. C., Tiao, G., Cummings, B. B., Alföldi, J., Wang, Q., & MacArthur, D. G. (2019). Variation across 141,456 human exomes and genomes reveals the spectrum of loss‐of‐function intolerance across human protein‐coding genes. bioRxiv, 531210. 10.1101/531210.

[mgg31709-bib-0015] Kassem, H. S., Azer, R. S., Ayad, M. S., Moharem‐Elgamal, S., Magdy, G., Elguindy, A., Cecchi, F., Olivotto, I., & Yacoub, M. H. (2013). Early results of sarcomeric gene screening from the Egyptian National BA‐HCM Program. Journal of Cardiovascular Translational Research, 6(1), 65–80. 10.1007/s12265-012-9425-0 23233322 PMC3546296

[mgg31709-bib-0016] Keeling, P. J., Gang, Y., Smith, G., Seo, H., Bent, S. E., Murday, V., Caforio, A. L., & McKenna, W. J. (1995). Familial dilated cardiomyopathy in the United Kingdom. British Heart Journal, 73(5), 417–421. 7786655 10.1136/hrt.73.5.417 PMC483856

[mgg31709-bib-0017] Lek, M., Karczewski, K. J., Minikel, E. V., Samocha, K. E., Banks, E., Fennell, T., O’Donnell‐Luria, A. H., Ware, J. S., Hill, A. J., Cummings, B. B., Tukiainen, T., Birnbaum, D. P., Kosmicki, J. A., Duncan, L. E., Estrada, K., Zhao, F., Zou, J., Pierce‐Hoffman, E., Berghout, J., … MacArthur, D. G. (2016). Analysis of protein‐coding genetic variation in 60,706 humans. Nature, 536, 285. 10.1038/nature19057. https://www.nature.com/articles/nature19057#supplementary‐information 27535533 PMC5018207

[mgg31709-bib-0018] Li, H., Handsaker, B., Wysoker, A., Fennell, T., Ruan, J., Homer, N., Marth, G., Abecasis, G., & Durbin, R. (2009). The Sequence Alignment/Map format and SAMtools. Bioinformatics (Oxford, England), 25(16), 2078–2079. 10.1093/bioinformatics/btp352 PMC2723002 19505943

[mgg31709-bib-0019] Maddirevula, S., Alzahrani, F., Al‐Owain, M., Al Muhaizea, M. A., Kayyali, H. R., AlHashem, A., Rahbeeni, Z., Al‐Otaibi, M., Alzaidan, H. I., Balobaid, A., El Khashab, H. Y., Bubshait, D. K., Faden, M., Yamani, S. A., Dabbagh, O., Al‐Mureikhi, M., Jasser, A. A., Alsaif, H. S., Alluhaydan, I., … Alkuraya, F. S. (2019). Autozygome and high throughput confirmation of disease genes candidacy. Genetics in Medicine, 21(3), 736–742. 10.1038/s41436-018-0138-x 30237576 PMC6752307

[mgg31709-bib-0020] Marian, A. J. (2010). Hypertrophic cardiomyopathy: From genetics to treatment. European Journal of Clinical Investigation, 40(4), 360–369. 10.1111/j.1365-2362.2010.02268.x 20503496 PMC2903630

[mgg31709-bib-0021] Mazzarotto, F., Tayal, U., Buchan, R. J., Midwinter, W., Wilk, A., Whiffin, N., Govind, R., Mazaika, E., de Marvao, A., Dawes, T. J. W., Felkin, L. E., Ahmad, M., Theotokis, P. I., Edwards, E., Ing, A. Y., Thomson, K. L., Chan, L. L. H., Sim, D., Baksi, A. J., … Walsh, R. (2020). Reevaluating the genetic contribution of monogenic dilated cardiomyopathy. Circulation, 141(5), 387–398. 10.1161/circulationaha.119.037661 31983221 PMC7004454

[mgg31709-bib-0022] McKenna, A., Hanna, M., Banks, E., Sivachenko, A., Cibulskis, K., Kernytsky, A., Garimella, K., Altshuler, D., Gabriel, S., Daly, M., & DePristo, M. A. (2010). The Genome Analysis Toolkit: a MapReduce framework for analyzing next‐generation DNA sequencing data. Genome Research, 20(9), 1297–1303. 10.1101/gr.107524.110 20644199 PMC2928508

[mgg31709-bib-0023] McKenna, W. J., Maron, B. J., & Thiene, G. (2017). Classification, Epidemiology, and Global Burden of Cardiomyopathies. Circulation Research, 121(7), 722–730. 10.1161/CIRCRESAHA.117.309711 28912179

[mgg31709-bib-0024] McNally, E. M., Barefield, D. Y., & Puckelwartz, M. J. (2015). The genetic landscape of cardiomyopathy and its role in heart failure. Cell Metabolism, 21(2), 174–182. 10.1016/j.cmet.2015.01.013 25651172 PMC4331062

[mgg31709-bib-0025] Nicora, G., Limongelli, I., Gambelli, P., Memmi, M., Malovini, A., Mazzanti, A., Napolitano, C., Priori, S., & Bellazzi, R. (2018). CardioVAI: An automatic implementation of ACMG‐AMP variant interpretation guidelines in the diagnosis of cardiovascular diseases. Human Mutation, 39(12), 1835–1846. 10.1002/humu.23665 30298955

[mgg31709-bib-0026] Pedersen, B. S., Layer, R. M., & Quinlan, A. R. (2016). Vcfanno: Fast, flexible annotation of genetic variants. Genome Biology, 17(1), 118. 10.1186/s13059-016-0973-5 27250555 PMC4888505

[mgg31709-bib-0027] Pua, C. J., Bhalshankar, J., Miao, K., Walsh, R., John, S., Lim, S. Q., Chow, K., Buchan, R., Soh, B. Y., Lio, P. M., Lim, J., Schafer, S., Lim, J. Q., Tan, P., Whiffin, N., Barton, P. J., Ware, J. S., & Cook, S. A. (2016). Development of a comprehensive sequencing assay for inherited cardiac condition genes. Journal of Cardiovascular Translational Research, 9(1), 3–11. 10.1007/s12265-016-9673-5 26888179 PMC4767849

[mgg31709-bib-0029] Rentzsch, P., Witten, D., Cooper, G. M., Shendure, J., & Kircher, M. (2019). CADD: predicting the deleteriousness of variants throughout the human genome. Nucleic Acids Research, 47(D1), D886–D894. 10.1093/nar/gky1016 30371827 PMC6323892

[mgg31709-bib-0030] Retterer, K., Juusola, J., Cho, M. T., Vitazka, P., Millan, F., Gibellini, F., Vertino‐Bell, A., Smaoui, N., Neidich, J., Monaghan, K. G., McKnight, D., Bai, R., Suchy, S., Friedman, B., Tahiliani, J., Pineda‐Alvarez, D., Richard, G., Brandt, T., Haverfield, E., … Bale, S. (2016). Clinical application of whole‐exome sequencing across clinical indications. Genetics in Medicine, 18(7), 696–704. 10.1038/gim.2015.148 26633542

[mgg31709-bib-0031] Richards, S., Aziz, N., Bale, S., Bick, D., Das, S., Gastier‐Foster, J., Grody, W. W., Hegde, M., Lyon, E., Spector, E., Voelkerding, K., & Rehm, H. L. (2015). Standards and guidelines for the interpretation of sequence variants: a joint consensus recommendation of the American College of Medical Genetics and Genomics and the Association for Molecular Pathology. Genetics in Medicine, 17(5), 405–424. 10.1038/gim.2015.30 25741868 PMC4544753

[mgg31709-bib-0032] Ruffier, M., Kähäri, A., Komorowska, M., Keenan, S., Laird, M., Longden, I., Proctor, G., Searle, S., Staines, D., Taylor, K., Vullo, A., Yates, A., Zerbino, D., & Flicek, P. (2017). Ensembl core software resources: storage and programmatic access for DNA sequence and genome annotation. Database (Oxford), 2017(1). 10.1093/database/bax020 PMC5467575 28365736

[mgg31709-bib-0033] Scott, E. M., Halees, A., Itan, Y., Spencer, E. G., He, Y., Azab, M. A., Gabriel, S. B., Belkadi, A., Boisson, B., Abel, L., Clark, A. G., Alkuraya, F. S., Casanova, J.‐L., & Gleeson, J. G. (2016). Characterization of Greater Middle Eastern genetic variation for enhanced disease gene discovery. Nature Genetics, 48(9), 1071–1076. 10.1038/ng.3592 27428751 PMC5019950

[mgg31709-bib-0035] Taliun, D., Harris, D. N., Kessler, M. D., Carlson, J., Szpiech, Z. A., Torres, R., & Abecasis, G. R. (2019). Sequencing of 53,831 diverse genomes from the NHLBI TOPMed Program. bioRxiv, 563866. 10.1101/563866 PMC7875770 33568819

[mgg31709-bib-0036] The 1000 Genomes Project Consortium . (2015). A global reference for human genetic variation. Nature, 526, 68–74. 10.1038/nature15393 26432245 PMC4750478

[mgg31709-bib-0037] Tobita, T., Nomura, S., Fujita, T., Morita, H., Asano, Y., Onoue, K., Ito, M., Imai, Y., Suzuki, A., Ko, T., Satoh, M., Fujita, K., Naito, A. T., Furutani, Y., Toko, H., Harada, M., Amiya, E., Hatano, M., Takimoto, E., … Komuro, I. (2018). Genetic basis of cardiomyopathy and the genotypes involved in prognosis and left ventricular reverse remodeling. Scientific Reports, 8(1), 1998. 10.1038/s41598-018-20114-9 29386531 PMC5792481

[mgg31709-bib-0038] Whiffin, N., Walsh, R., Govind, R., Edwards, M., Ahmad, M., Zhang, X., Tayal, U., Buchan, R., Midwinter, W., Wilk, A. E., Najgebauer, H., Francis, C., Wilkinson, S., Monk, T., Brett, L., O’Regan, D. P., Prasad, S. K., Morris‐Rosendahl, D. J., Barton, P. J. R., … Cook, S. A. (2018). CardioClassifier: disease‐ and gene‐specific computational decision support for clinical genome interpretation. Genetics in Medicine, 20(10), 1246–1254. 10.1038/gim.2017.258 29369293 PMC6558251

[mgg31709-bib-0039] Yacoub, M. H. (2014). Decade in review–cardiomyopathies: Cardiomyopathy on the move. Nature Reviews Cardiology, 11(11), 628–629. 10.1038/nrcardio.2014.157 25267422

