# Supplementary material for: Genetic evaluation of cardiomyopathies in Qatar identifies enrichment of pathogenic sarcomere gene variants and possible founder disease mutations in the Arabs
Source: Mol Genet Genomic Med. 2021 Jun 17;9(7):e1709. doi: 10.1002/mgg3.1709 (PMC8372065; doi:10.1002/mgg3.1709)

**Supplementary Table 1.**

Targeted genes of the Trusight Cardio sequencing kit in the study.

Genes highlighted in yellow are the HCM asscoiated genes. Genes highlighted in blue are the putative DCM genes, while genes highlighted in green are the overlapped genes between HCM and DCM analysis, since they are HCM asscoiated genes and DCM putative genes.

**Supplementary Table 3.**

Accession numbers of the vaiants deposited in ClinVar will be provided.

**Supplementary Figure 1.**

Pedigrees of the informative HCM and DCM families, and their detected genotypes. +/+ genotype is a homozygous for the reference allele, +/- is a hetrozygous for the alternative allele, -/- is a homozygous for the alternative allele.

**Supplementary Figure 2.**

IGV screen shots of the novel variants in HCM cases (screen shots 1 to 4), and DCM cases (screen shots 5 and 6).

1.- HCM3, Position:179666987, *TTN*, c.170_172delCCT, p.Ser57del


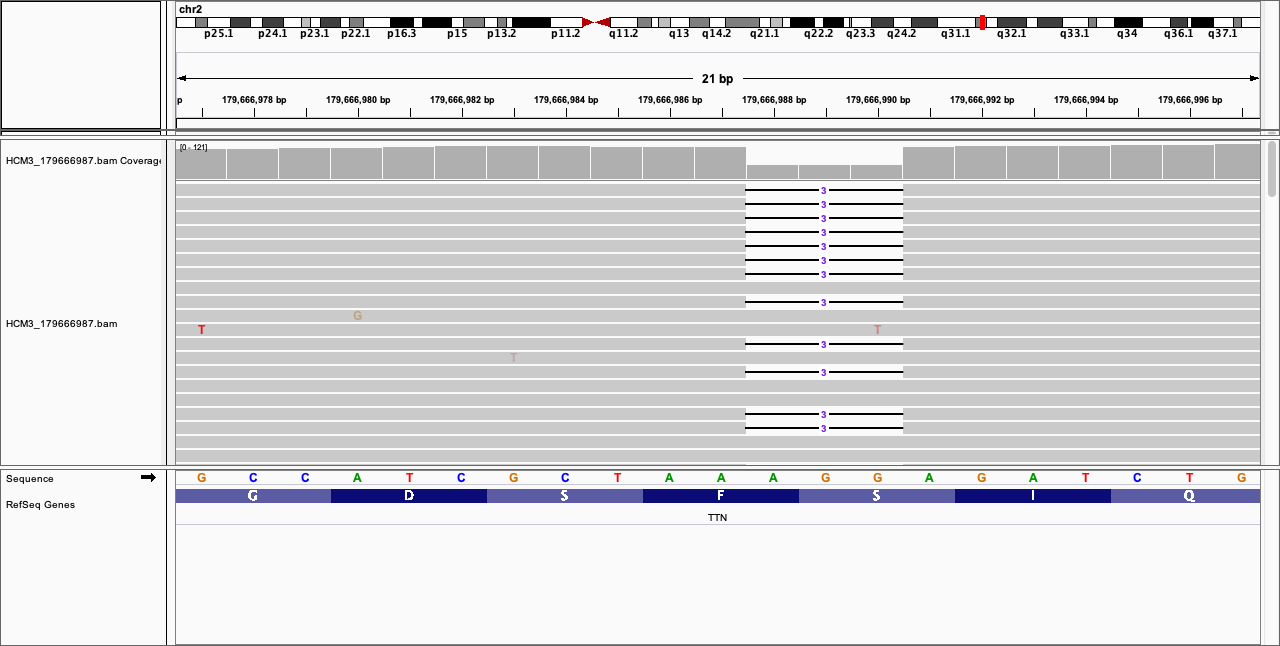


2.- HCM 99, Position:47355287, *MYBPC3*, c.3009_3010delTC, p.Gln1004fs


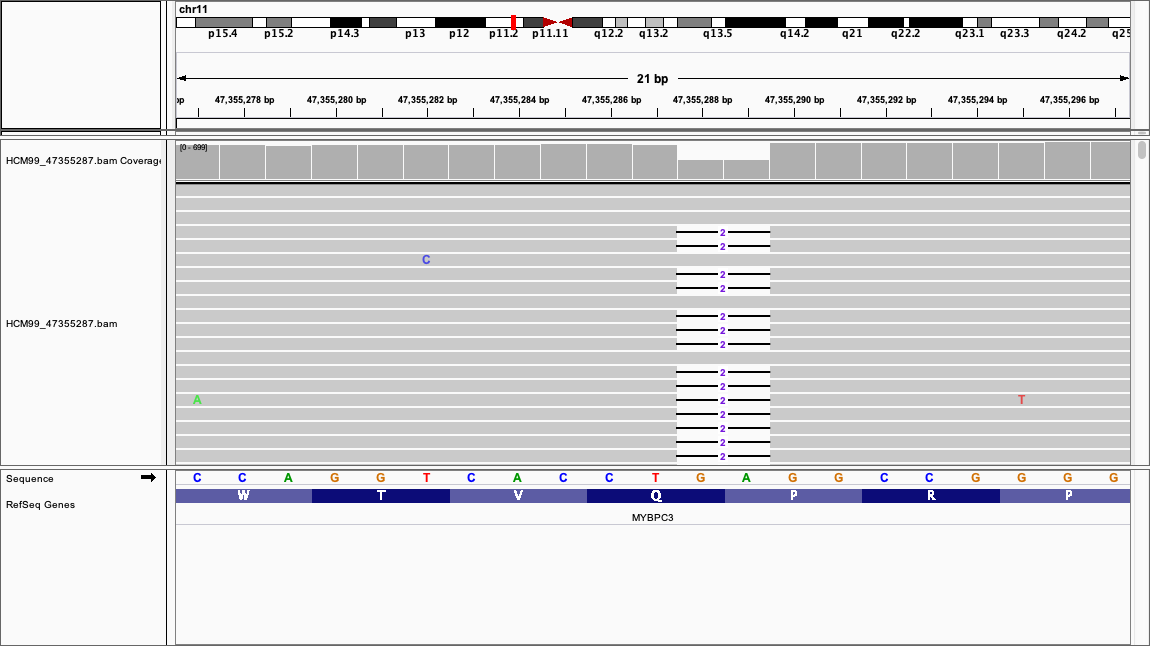


3.- HCM51, Position:47355287, *MYBPC3*, c.3009_3010delTC p.Gln1004fs


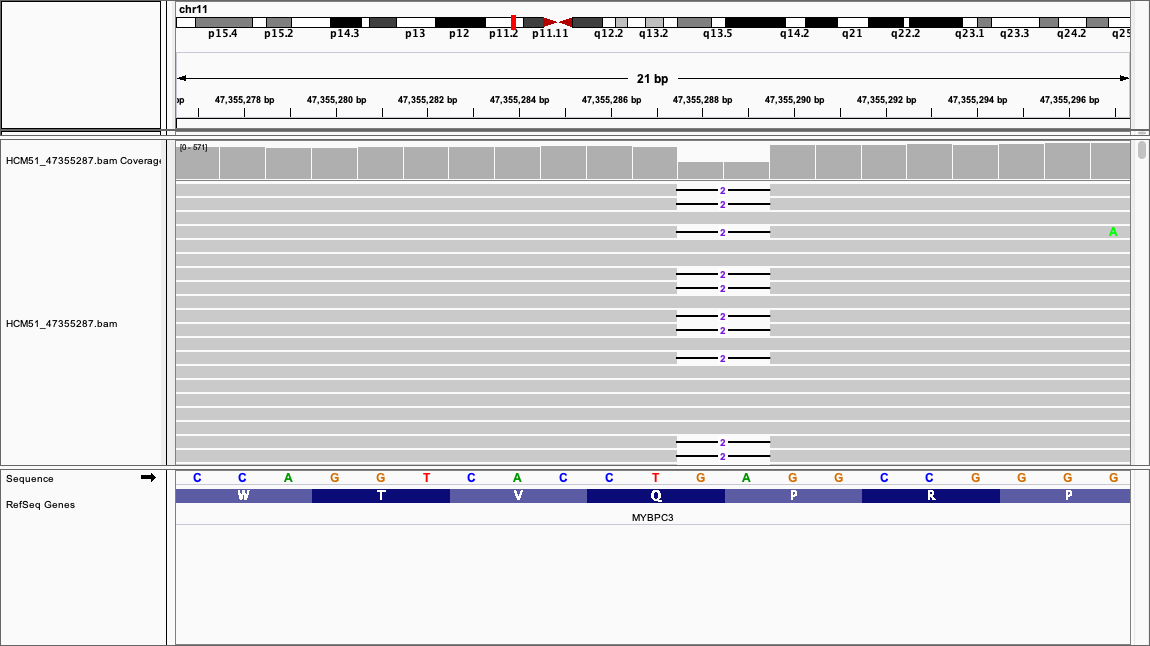


4.- HCM5, Position:47373030, *MYBPC3*, c.51dupG, p.Ser18fs


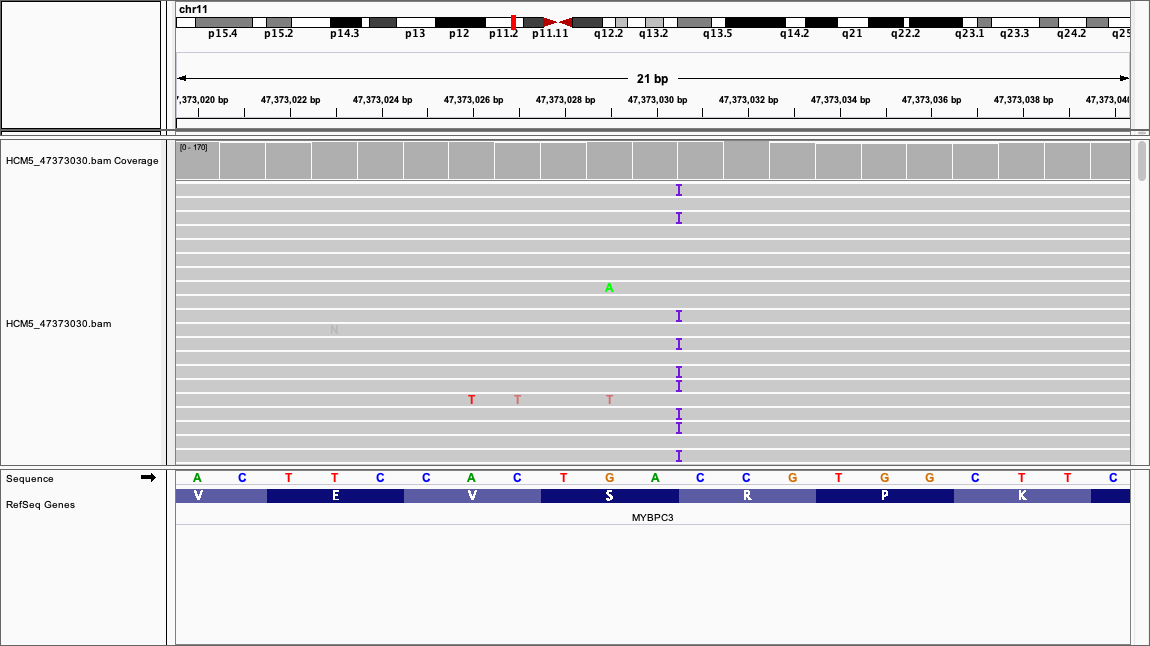


5- DCM11, Position:179437023, *TTN,* c.73828_7385delTCTGTGAA, p.Val24611fs


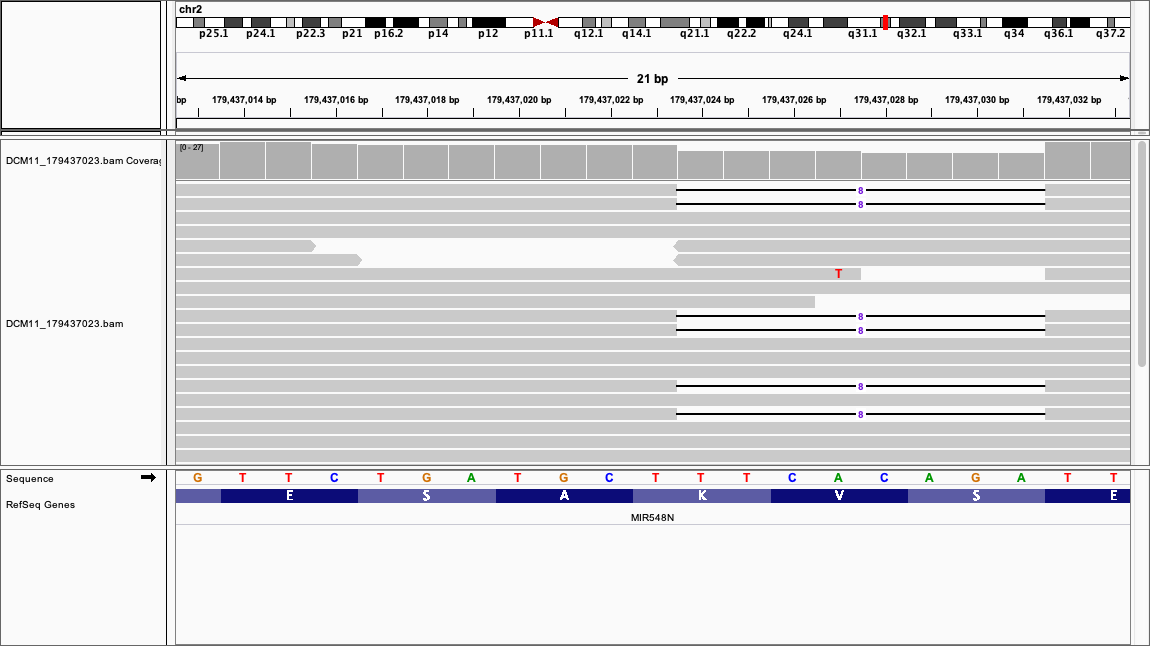


6- DCM 5, Position:179469488, *TTN,* c.54327delG, p.Lys18110fs


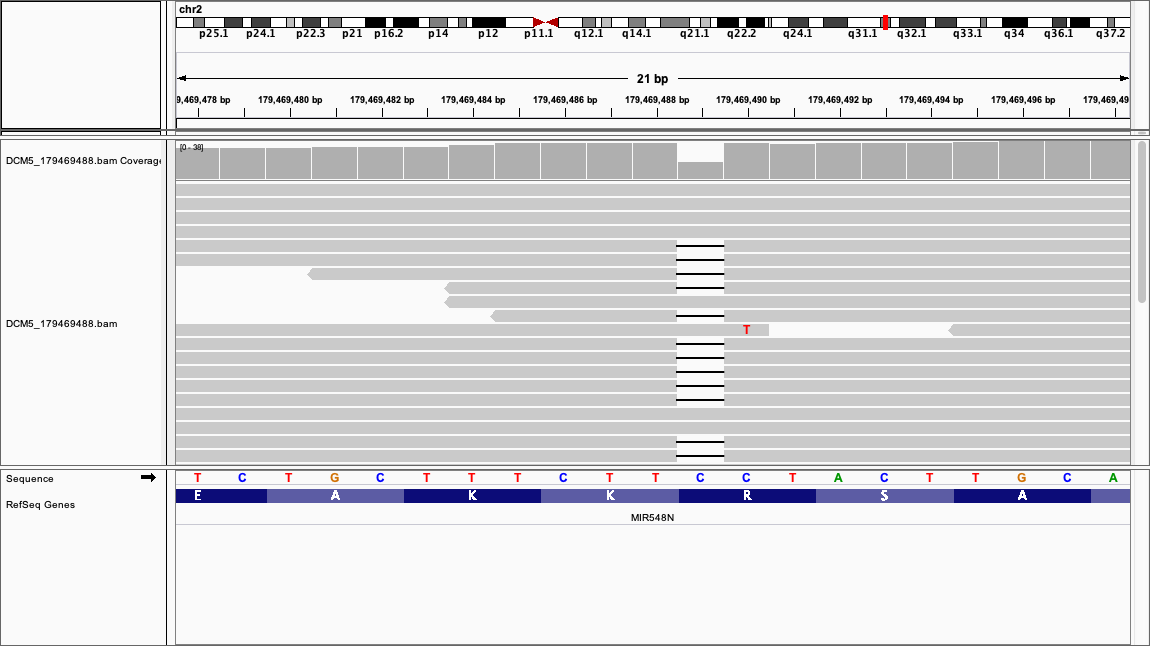

Supplement: Supplementary file 1 — Table S1‐Fig S1‐S2 [file MGG3-9-e1709-s002.docx]
